# Protein associations and protein–metabolite interactions with depressive symptoms and the p-factor

**DOI:** 10.1038/s41398-025-03362-y

**Published:** 2025-04-06

**Authors:** Alyce M. Whipp, Gabin Drouard, Richard J. Rose, Lea Pulkkinen, Jaakko Kaprio

**Affiliations:** 1 Institute for Molecular Medicine Finland (FIMM), HiLIFE, University of Helsinki, Helsinki, Finland; 2 Department of Psychological & Brain Sciences, Indiana University, Bloomington, IN USA; 3 Department of Psychology, University of Jyväskylä, Jyväskylä, Finland

**Keywords:** Human behaviour, Biomarkers

## Abstract

Despite increasing mental health problems among young people, few studies have examined associations between plasma proteins and mental health. Interactions between proteins and metabolites in association with mental health problems remain underexplored. In 730 twins, we quantified associations between plasma proteins measured at age 22 with 21 indicators of either depressive symptoms or the p-factor and tested for interactions with metabolites. Symptoms were collected from questionnaires and interviews completed by different raters (e.g., self-report, teachers) through adolescence to young adulthood (12 to 22 years). We found 47 proteins associated with depressive symptoms or the p-factor (FDR < 0.2), 9 being associated with both. Two proteins, contactin-1 and mast/stem cell growth factor receptor kit, positively interacted with valine levels in explaining p-factor variability. Our study demonstrates strong associations between plasma proteins and mental health and provides evidence for proteome–metabolome interactions in explaining higher levels of mental health problems.

## Introduction

With mental health problems on the rise [1], and the poor success rates of existing treatments for mental health disorders like depression [2], we need to improve our understanding of the biological mechanisms involved in these disorders. The omics revolution has brought the ability to examine mechanisms of a health problem from the genomic and epigenomic levels, and more recently from the gut microbiome, metabolomic and proteomic levels. As these omics technologies are now being increasingly used in investigations of mental health [3–5], we are starting to have more pieces of the puzzle. However, these disorders are heterogeneous and complex and using multi-omic approaches may bring more clarity.

The proteome and metabolome refer to the total set of proteins and metabolites, respectively, within a biological system, commonly measured in blood. While the proteome and metabolome may differ in nature, both are influenced by genetic and environmental factors [6, 7] and both are dynamic, making them optimal resources for biomarker investigations. Combining proteomic data with metabolomic data allows for a refined understanding of the link between the beginning of the biologic pathway (the genetics) and the end (the metabolomics) for a health problem, since the proteome is an intermediary. Additionally, the different levels of investigation that multi-omics allow for could provide more clues about the lesser-known triggers to poor mental health such as inflammatory disorders, infections, or metabolic conditions [8, 9]. For example, in a recent multi-omic investigation on body mass index (BMI) by our group, combining proteomics, metabolomics, transcriptomics, and polygenic risk scores allowed us to show that the associations between plasma proteins and changes in BMI during adolescence were characterized by common metabolic etiologies [10]. To our knowledge, multi-omic approaches are scarce. We are unaware of published studies examining interactions between the proteome and metabolome. To date, even single-omic approaches to mental health have been limited in the literature and have involved a single outcome, as has been demonstrated in two recent studies using the Strengths and Difficulties Questionnaire (SDQ) [11] and the p-factor [12]. Furthermore, while there have been a few large-scale studies that have investigated mental health with proteomics data, using for example UK Biobank data [13], these studies were performed on older adults only. Whether the association of the plasma proteome with depressive symptoms and the p-factor differs by age, or by rater, has not been investigated to the best of our knowledge.

Previously, our group has investigated the metabolomics of mental health, including depressive symptoms and aggressive behavior, in young adult Finnish twins. We found two branched-chain amino acids (BCAAs) -- valine and leucine -- that were (negatively) associated with depressive symptoms in our FinnTwin12 cohort [14]. The trend in these associations were consistent in meta-analysis across multiple ages, raters, and instruments of depressive symptoms. Additionally, we found a ketone body (3-hydroxybutyrate) that was negatively associated with aggressive behavior: that finding was suggestively replicated in a Dutch twin sample [15]. Biomarkers for the p-factor were also sought, but no additional metabolites from this analysis were identified [14]. The p-factor, or psychopathology factor, initially characterized by Caspi et al. [16], aggregates internalizing disorders (such as depression), externalizing disorders (such as aggression/conduct problems), and thought disorders (such as schizophrenia) into a higher-order combined dimension, which has shown a common underlying genetics [17], despite the heterogeneity in the disorders. Our group has recently acquired proteomic data on the same cohort as the metabolomic analyses were performed, and thus have the unique opportunity to use a multi-omic approach to enrich our previous findings.

**Fig. 1:**
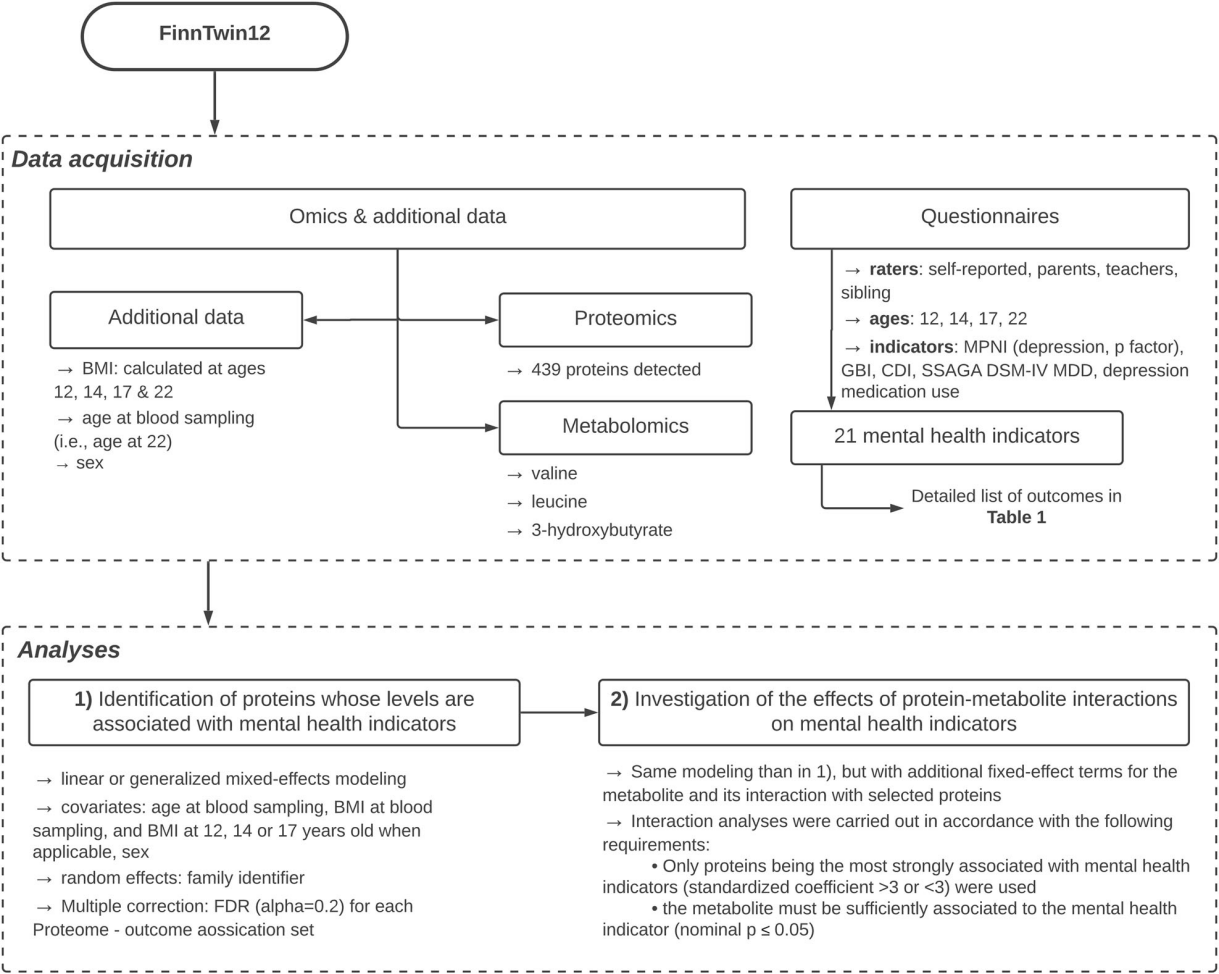
Overall study workflow.

Thus, we aimed to investigate, in an exploratory way, proteomic associations with depressive symptoms and the p-factor in our FinnTwin12 cohort (Fig. 1). We used a total of 21 outcomes that describe depressive symptoms (including measures of major depressive disorder (MDD) and summary scores of general depressive symptoms) and the p-factor (combining internalizing and externalizing problems); these were obtained from different raters and were reported at different ages, from childhood to young adulthood. Furthermore, for significant protein associations, we aimed to investigate possible protein–metabolite interactions with the significant BCAA and 3-hydroxybutyrate metabolite associations found in the previous FinnTwin12 investigations. This study plan was pre-registered at Open Science Framework (OSF) in May 2023 (osf.io/kc9hw).

## Results

**Fig. 2:**
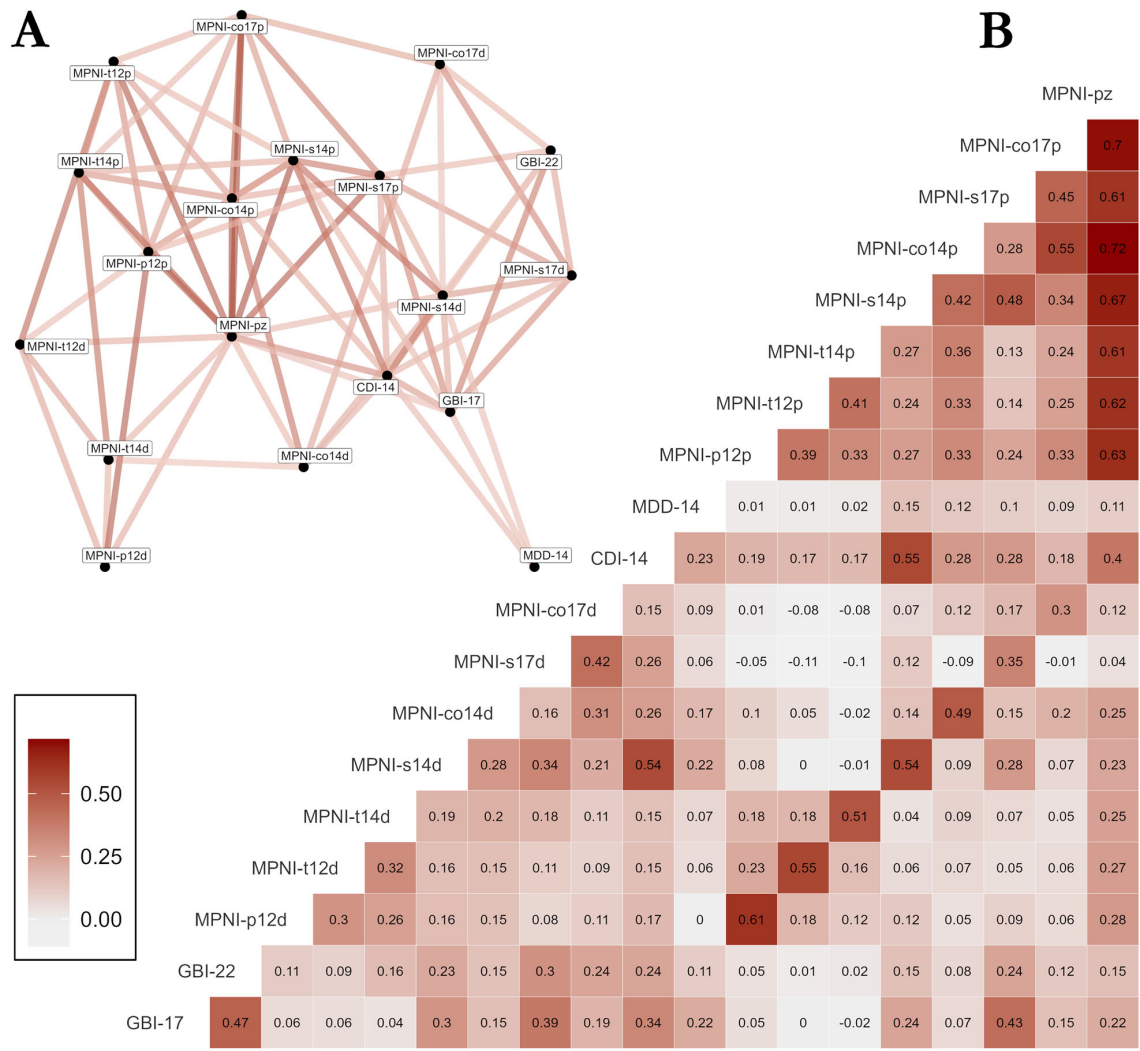
Pairwise Pearson correlations between continuous mental health indicators. Pairwise Pearson correlations are presented in two different formats: **A** a graph connecting mental health indicators if the pairwise Pearson correlation exceeds 0.2 in absolute value, and **B** a correlation matrix.

**Fig. 3:**
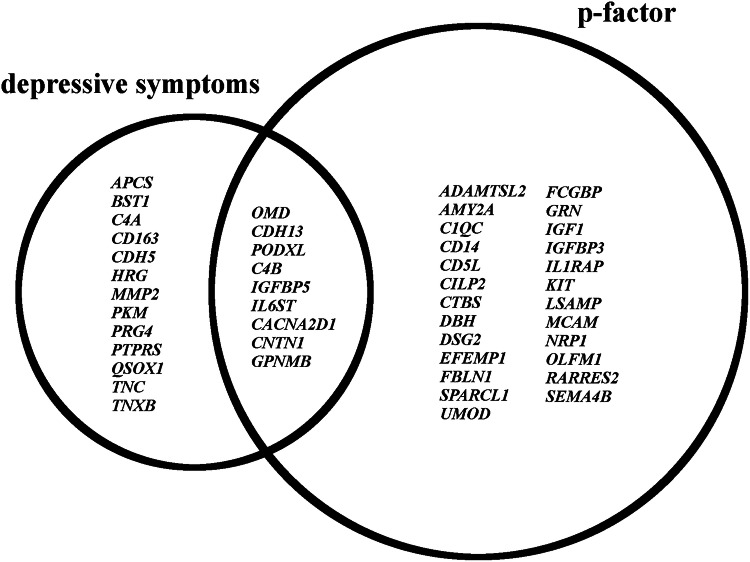
Venn diagram of protein-coding genes. The genes encoding the identified proteins were partitioned according to whether their encoded proteins were associated only with depressive symptoms (CDI, MPNId, GBI), only with the p-factor (across raters and age groups), or with both. See Table 2 for protein descriptions.

We first modeled the 21 different mental health indicators (Table 1) as dependent variables and age at blood sampling, sex, and BMI as covariates. A description of the mental health indicators and their intricate relationships is presented in Table 1 and Fig. 2, respectively. We found 68 significant associations between proteins and mental health indicators at an FDR ≤ 0.2 level (Table 2; 628 associations with nominal p < 0.05, see Supplemental Table 1). Altogether, significant associations involved 47 unique proteins, of which 17 were associated with two or more mental health indicators. Of the 68 associations, 24 were with depressive symptom variables (CDI-14 (n = 1; effect size −0.65), GBI-22 (n = 4; effect sizes ranging −0.57 to 0.61), and MPNI-s14d (n = 19; effect sizes ranging −0.05 to 0.05)) and 44 with p-factor variables (MPNI-pz (n = 11; effect sizes ranging −0.18 to 0.13), MPNI-s17p (n = 11; effect sizes ranging −1.34 to 1.33), and MPNI-s14p (n = 22; effect sizes ranging −0.99 to 1.08)). Some proteins were significantly associated with both depressive symptoms and p-factor variables (9 proteins), while others were significant only with depressive symptoms (13 proteins) or the p-factor (25 proteins)(Fig. 3). Additionally, 58 of the associations were negative, 10 positive. The strongest associations were found with the p-factor, with the highest statistical significance between: IgGFc-binding protein with MPNI-pz (t-value = −4.81; nominal p = 1.9e-6) and MPNI-co14d (t-value = −4.0; nominal p = 5.9e-5), complement C4-b with MPNI-s14d (t-value = 4.1; nominal p = 4.4e-5), Fibulin-1 with MPNI-s17d (t-value = −4.1; nominal p = 5.0e-5), and Monocyte Differentiation Antigen CD14 with MPNI-pz (t-value = 4.0; nominal p = 7.5e-5) (Table 2).

**Table 1:** Description of 21 mental health indicators in participants with proteomic data.

Variable name	Instrument/Scale	Age at report	Rater	N	Mean	SD	Range	Skewness	Kurtosis
MPNI-p12d	MPNI - depression	12	parent(s)	694	0.75	0.4	0–2.4	0.8	0.5
MPNI-t12d	MPNI - depression	12	teacher	704	0.64	0.5	0–2.8	1.0	1.2
MPNI-t14d	MPNI - depression	14	teacher	565	0.53	0.5	0–2.4	1.0	1.0
MPNI-s14d	MPNI - depression	14	self-report	692	0.66	0.4	0–2.4	0.6	0.3
MPNI-co14d	MPNI - depression	14	co-twin	695	0.61	0.4	0–2.2	0.6	0.2
MPNI-s17d	MPNI - depression	17	self-report	655	0.73	0.7	0–3.0	0.8	0.1
MPNI-co17d	MPNI - depression	17	co-twin	650	0.67	0.6	0–3.0	0.7	0.0
CDI-14	CDI	14	self-report	704	6.25	5.1	0–40	1.7	5.2
MDD-14	SSAGA DSM-IV MDD lifetime depressive symptoms	14	self (interview)	726	0.23	0.61	0–5.0	3.63	18.1
MDD-22 (*)	SSAGA DSM-IV MDD diagnosis, Y/N	22	self (interview)	730	cases: 14%				
GBI-17	GBI	17	self-report	663	4.90	4.8	0–30	1.6	3.3
GBI-22	GBI	22	self-report	723	4.49	4.6	0–29	1.8	4.5
medication-22d (*)	Using depression medication, Y/N	22	self (interview)	730	cases: 3%				
MPNI-p12p	MPNI - pfactor	12	parent(s)	688	16.22	7.9	0–49	0.8	0.8
MPNI-t12p	MPNI - pfactor	12	teacher	688	15.47	11.4	0–61	1.0	0.9
MPNI-t14p	MPNI - pfactor	14	teacher	554	11.17	9.3	0–50	1.4	1.7
MPNI-s14p	MPNI - pfactor	14	self-report	682	16.38	6.8	2–44	0.6	0.5
MPNI-co14p	MPNI - pfactor	14	co-twin	675	16.53	8.2	1–46	0.6	0.3
MPNI-s17p	MPNI - pfactor	17	self-report	631	19.22	6.5	3–41	0.4	0.0
MPNI-co17p	MPNI - pfactor	17	co-twin	627	19.13	7.8	3–46	0.4	−0.3
MPNI-pz	MPNI - pfactor (z-scored score of all 7 individual pfactor variables)	-	-	721	−0.03	1.0	−2.2–4.3	0.6	0.4

The 21 mental health indicators were calculated from questionnaires completed by different raters at different ages during adolescence and young adulthood. The number of participants with proteomic data was 730. The symbol (*) denotes categorical indicators; others are continuous. *CDI* Children’s Depression Inventory, *DSM* Diagnostic and Statistical Manual of Mental Disorders, *GBI* General Behavior Inventory, *MDD* MPNI: Multidimensional Peer Nomination Inventory, *N* Number of participants with proteomic data who also had non-missing information for each mental health indicator, *SD* standard deviation. *SSAGA* Semi-Structured Assessment for the Genetics of Alcoholism.

**Table 2:** Associations between protein abundance and mental health indicators.

	Protein description			Fixed-effect coefficient			P-value		
Outcome	Protein	Genes	Description	Estimate	se	t/z.values	nominal	FDR	Bonferroni
CDI-14	Q99983	OMD	Osteomodulin	−0.65	0.2	−3.6	3.4E-04	1.5E-01	1.5E-01
GBI-22	P55290	CDH13	Cadherin-13	−0.56	0.2	−3.2	1.4E-03	2.0E-01	6.0E-01
GBI-22	P33151	CDH5	Cadherin-5	−0.57	0.2	−3.2	1.3E-03	2.0E-01	5.5E-01
GBI-22	Q92954	PRG4	Proteoglycan 4	0.61	0.2	3.1	1.8E-03	2.0E-01	7.9E-01
GBI-22	Q86VB7	CD163	Scavenger receptor cysteine-rich type 1 protein M130	0.58	0.2	3.3	9.1E-04	2.0E-01	4.0E-01
MPNI-pz	Q12860	CNTN1	Contactin-1	−0.11	0.0	−3.1	2.2E-03	1.4E-01	9.8E-01
MPNI-pz	Q14126	DSG2	Desmoglein-2	−0.10	0.0	−2.8	4.6E-03	1.8E-01	1
MPNI-pz	P09172	DBH	Dopamine beta-hydroxylase	−0.13	0.0	−3.6	3.9E-04	4.8E-02	1.7E-01
MPNI-pz	P23142	FBLN1	Fibulin-1	−0.10	0.0	−3.0	3.1E-03	1.7E-01	1
MPNI-pz	Q9Y6R7	FCGBP	IgGFc-binding protein	−0.18	0.0	−4.8	1.9E-06	8.3E-04	8.3E-04
MPNI-pz	P08571	CD14	Monocyte differentiation antigen CD14	0.13	0.0	4.0	7.5E-05	1.6E-02	3.3E-02
MPNI-pz	Q99784	OLFM1	Noelin	−0.10	0.0	−2.9	3.5E-03	1.7E-01	1
MPNI-pz	Q99983	OMD	Osteomodulin	−0.10	0.0	−3.2	1.3E-03	9.8E-02	5.9E-01
MPNI-pz	Q99969	RARRES2	Retinoic acid receptor responder protein 2	0.11	0.0	3.5	4.4E-04	4.8E-02	1.9E-01
MPNI-pz	Q14515	SPARCL1	SPARC-like protein 1	−0.10	0.0	−2.9	4.0E-03	1.8E-01	1
MPNI-pz	P07911	UMOD	Uromodulin	−0.11	0.0	−3.3	9.3E-04	8.1E-02	4.1E-01
MPNI-s14p	Q86TH1	ADAMTSL2	ADAMTS-like protein 2	−0.79	0.3	−3.1	2.3E-03	1.3E-01	1
MPNI-s14p	P55290	CDH13	Cadherin-13	−0.87	0.3	−3.3	1.1E-03	9.3E-02	4.6E-01
MPNI-s14p	Q8IUL8	CILP2	Cartilage intermediate layer protein 2	−0.70	0.3	−2.8	5.9E-03	1.6E-01	1
MPNI-s14p	P43121	MCAM	Cell surface glycoprotein MUC18	−0.70	0.3	−2.7	7.5E-03	1.6E-01	1
MPNI-s14p	P02747	C1QC	Complement C1q subcomponent subunit C	−0.77	0.3	−3.0	3.2E-03	1.5E-01	1
MPNI-s14p	P0C0L5	C4B	Complement C4-B	1.08	0.3	4.1	4.4E-05	1.9E-02	1.9E-02
MPNI-s14p	Q14126	DSG2	Desmoglein-2	−0.69	0.3	−2.8	6.0E-03	1.6E-01	1
MPNI-s14p	Q9Y6R7	FCGBP	IgGFc-binding protein	−0.75	0.3	−2.7	7.3E-03	1.6E-01	1
MPNI-s14p	P05019	IGF1	Insulin-like growth factor I	−0.74	0.3	−2.7	7.0E-03	1.6E-01	1
MPNI-s14p	P17936	IGFBP3	Insulin-like growth factor-binding protein 3	−0.78	0.3	−2.9	4.1E-03	1.5E-01	1
MPNI-s14p	P24593	IGFBP5	Insulin-like growth factor-binding protein 5	−0.72	0.3	−2.8	5.8E-03	1.6E-01	1
MPNI-s14p	Q9NPH3	IL1RAP	Interleukin-1 receptor accessory protein	−0.92	0.3	−3.4	8.2E-04	9.1E-02	3.6E-01
MPNI-s14p	P40189	IL6ST	Interleukin-6 receptor subunit beta	−0.93	0.3	−3.7	2.2E-04	3.3E-02	9.8E-02
MPNI-s14p	Q13449	LSAMP	Limbic system-associated membrane protein	−0.76	0.3	−2.9	4.2E-03	1.5E-01	1
MPNI-s14p	O14786	NRP1	Neuropilin-1	−0.70	0.3	−2.7	6.3E-03	1.6E-01	1
MPNI-s14p	Q99784	OLFM1	Noelin	−0.75	0.3	−2.9	3.7E-03	1.5E-01	1
MPNI-s14p	O00592	PODXL	Podocalyxin	−0.85	0.3	−3.1	1.9E-03	1.2E-01	8.2E-01
MPNI-s14p	P28799	GRN	Progranulin	−0.68	0.3	−2.6	9.1E-03	1.9E-01	1
MPNI-s14p	Q9NPR2	SEMA4B	Semaphorin-4B	−0.73	0.3	−2.7	7.3E-03	1.6E-01	1
MPNI-s14p	Q14515	SPARCL1	SPARC-like protein 1	−0.99	0.3	−3.9	1.1E-04	2.4E-02	4.9E-02
MPNI-s14p	Q14956	GPNMB	Transmembrane glycoprotein NMB	−0.81	0.3	−3.1	1.9E-03	1.2E-01	8.4E-01
MPNI-s14p	P54289	CACNA2D1	Voltage-dependent calcium channel subunit alpha-2/delta-1	−0.67	0.3	−2.6	9.7E-03	1.9E-01	1
MPNI-s17p	Q12860	CNTN1	Contactin-1	−0.85	0.3	−3.2	1.6E-03	1.7E-01	7.1E-01
MPNI-s17p	Q12805	EFEMP1	EGF-containing fibulin-like extracellular matrix protein 1	−0.80	0.3	−3.1	2.0E-03	1.7E-01	8.6E-01
MPNI-s17p	P23142	FBLN1	Fibulin-1	−1.04	0.3	−4.1	5.0E-05	2.2E-02	2.2E-02
MPNI-s17p	Q9NPH3	IL1RAP	Interleukin-1 receptor accessory protein	−0.92	0.3	−3.3	1.1E-03	1.6E-01	4.7E-01
MPNI-s17p	P10721	KIT	Mast/stem cell growth factor receptor Kit	−0.97	0.3	−3.7	2.8E-04	6.2E-02	1.2E-01
MPNI-t12p	Q01459	CTBS	Di-N-acetylchitobiase	1.33	0.4	3.6	3.9E-04	1.7E-01	1.7E-01
MPNI-co14p	Q9Y6R7	FCGBP	IgGFc-binding protein	−1.34	0.3	−4.0	5.9E-05	2.6E-02	2.6E-02
MPNI-co17p	O43866	CD5L	CD5 antigen-like	−1.03	0.3	−3.3	1.2E-03	1.7E-01	5.3E-01
MPNI-co17p	P02747	C1QC	Complement C1q subcomponent subunit C	−1.07	0.3	−3.4	6.4E-04	1.7E-01	2.8E-01
MPNI-co17p	P04746	AMY2A	Pancreatic alpha-amylase	−0.99	0.3	−3.2	1.4E-03	1.7E-01	5.9E-01
MPNI-co17p	Q99969	RARRES2	Retinoic acid receptor responder protein 2	0.98	0.3	3.2	1.5E-03	1.7E-01	6.8E-01
MPNI-s14d	P08253	MMP2	72 kDa type IV collagenase	−0.04	0.0	−2.8	5.4E-03	1.5E-01	1
MPNI-s14d	Q10588	BST1	ADP-ribosyl cyclase/cyclic ADP-ribose hydrolase 2	−0.04	0.0	−2.7	6.1E-03	1.5E-01	1
MPNI-s14d	P55290	CDH13	Cadherin-13	−0.05	0.0	−3.4	8.5E-04	1.2E-01	3.7E-01
MPNI-s14d	P0C0L4	C4A	Complement C4-A	0.05	0.0	3.1	2.0E-03	1.2E-01	8.7E-01
MPNI-s14d	P0C0L5	C4B	Complement C4-B	0.04	0.0	2.8	5.9E-03	1.5E-01	1
MPNI-s14d	Q12860	CNTN1	Contactin-1	−0.05	0.0	−3.3	8.9E-04	1.2E-01	3.9E-01
MPNI-s14d	P04196	HRG	Histidine-rich glycoprotein	−0.04	0.0	−2.6	8.5E-03	2.0E-01	1
MPNI-s14d	P24593	IGFBP5	Insulin-like growth factor-binding protein 5	−0.05	0.0	−3.1	2.3E-03	1.2E-01	9.9E-01
MPNI-s14d	P40189	IL6ST	Interleukin-6 receptor subunit beta	−0.04	0.0	−3.0	3.0E-03	1.4E-01	1
MPNI-s14d	Q99983	OMD	Osteomodulin	−0.05	0.0	−3.2	1.7E-03	1.2E-01	7.4E-01
MPNI-s14d	O00592	PODXL	Podocalyxin	−0.05	0.0	−2.9	4.4E-03	1.4E-01	1
MPNI-s14d	P14618	PKM	Pyruvate kinase PKM	−0.04	0.0	−2.8	4.9E-03	1.4E-01	1
MPNI-s14d	Q13332	PTPRS	Receptor-type tyrosine-protein phosphatase S	−0.04	0.0	−2.9	3.8E-03	1.4E-01	1
MPNI-s14d	P02743	APCS	Serum amyloid P-component	0.05	0.0	2.9	3.6E-03	1.4E-01	1
MPNI-s14d	O00391	QSOX1	Sulfhydryl oxidase 1	−0.05	0.0	−2.8	4.6E-03	1.4E-01	1
MPNI-s14d	P24821	TNC	Tenascin	−0.05	0.0	−3.2	1.5E-03	1.2E-01	6.6E-01
MPNI-s14d	P22105	TNXB	Tenascin-X	−0.04	0.0	−2.8	4.5E-03	1.4E-01	1
MPNI-s14d	Q14956	GPNMB	Transmembrane glycoprotein NMB	−0.05	0.0	−3.2	1.7E-03	1.2E-01	7.4E-01
MPNI-s14d	P54289	CACNA2D1	Voltage-dependent calcium channel subunit alpha-2/delta-1	−0.05	0.0	−3.2	1.5E-03	1.2E-01	6.7E-01

Multiple correction was performed at the level of each mental health indicator using Bonferroni or FDR correction. Only associations for which FDR-adjusted p-value is below 0.2 are presented. *FDR* False Discovery Rate. *se* standard error.

We investigated which biological pathways may relate to the 47 proteins associated with mental health indicators by performing pathway analysis. A total of 19 pathways, as indexed by the Reactome database, were identified with FDR-corrected p-values below 0.05. The top 4 pathways were “Interleukin-6 signaling”, “Regulation of insulin-like growth factor (IGF) transport and uptake by insulin-like growth factor binding proteins (IGFBPs)”, “Interleukin-6 family signaling”, and “Post-translational protein phosphorylation”. We identified three additional pathways that also passed multiple testing correction: “Platelet degranulation”, “Response to elevated platelet cytosolic Ca2+”, and “Transfer of LPS from LBP carrier to CD14”. The remaining 12 pathways were only related to *KIT* function and were based on a single entity (*KIT*) that we identified in our analyses, and thus were of less interest. The results of the pathway analyses are available in the supplementary material (Table S2).

**Fig. 4:**
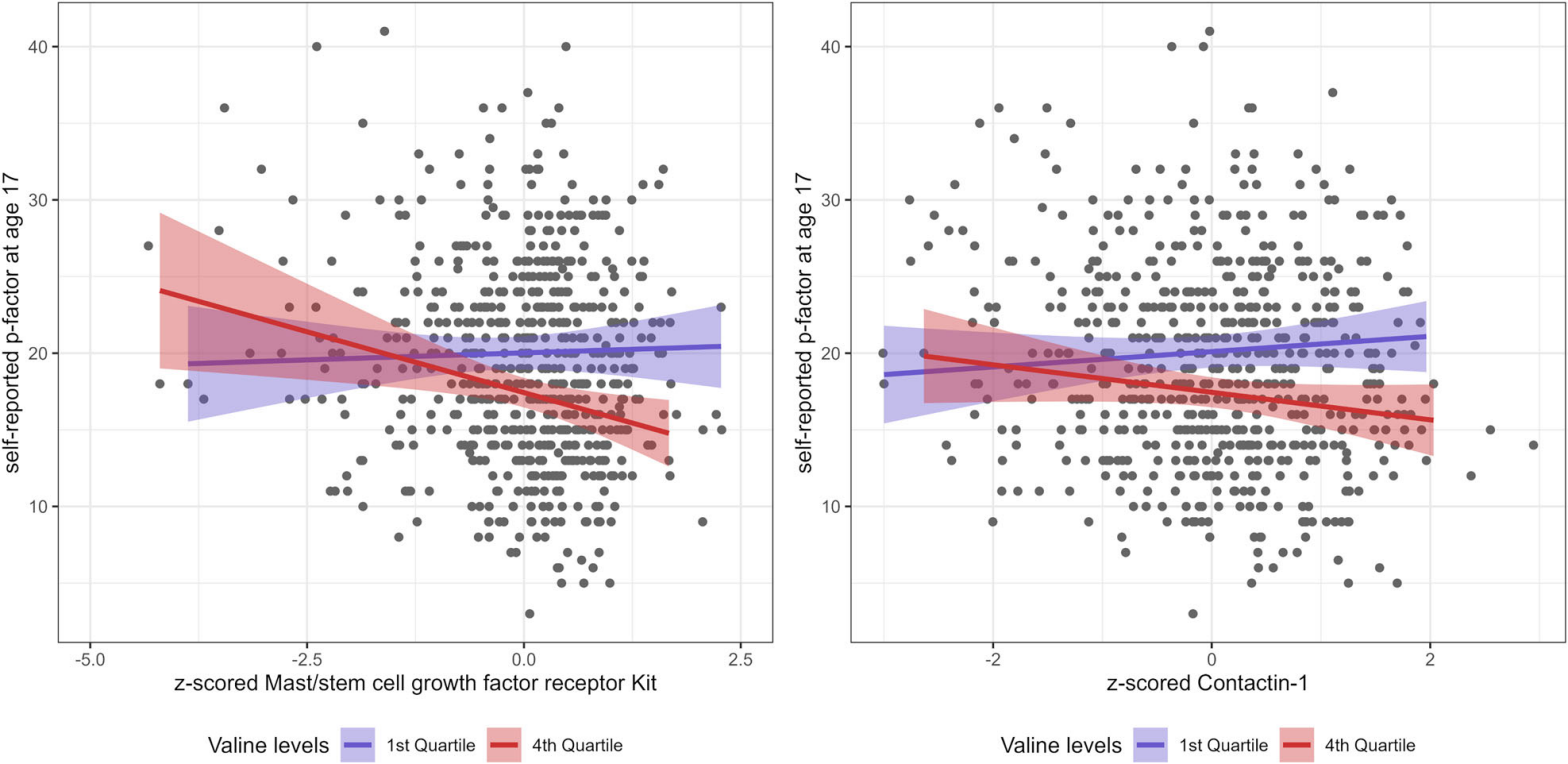
Graphical representation of significant protein–valine interactions. The plot represents Mast/stem cell growth factor receptor Kit and Contactin-1 z-scored levels (x axis) in relation to the self-reported p-factor score at age 17 (y axis). Participants having non-missing valine data are plotted. The y ~ x regression lines are shown for participants in the first or last quartile of valine levels.

In follow-up analyses, of the 47 proteins significantly associated with mental health indicators, those that were strongly associated with mental health (see Fig. 1) were investigated for protein–metabolite associations with mental health (the metabolite was also assessed to be highly significantly associated with mental health; see Fig. 1). Thus, seven interactions were tested. Of these, two protein–metabolite interactions were found with MPNI-s17p: Mast/stem cell growth factor receptor Kit and Valine (interaction term p = 0.01), and Contactin-1 and Valine (interaction term p = 0.04) (Table 3). To further illustrate the protein–metabolite interactions with mental health, the z-scored levels of these two valine-interacting proteins were then plotted with the self-reported p-factor values at age 17 (Fig. 4). Stratification by the first and last quartile of valine was performed to visualize shifts in protein associations with mental health. In participants with high levels of valine, the deleterious effects of the Contactin-1 or Mast/stem cell growth factor receptor kit on mental health are lower independent of valine’s effect alone.

**Table 3:** Interactions between selected protein abundance and metabolites.

	Protein			Protein estimate				Metabolite estimate				Protein:Metabolite interaction			
outcome	UniProt	Gene	Metabolite	coef	se	t-value	p	coef	se	t-value	p	coef	se	t-value	p
MPNI-s17p	P10721	KIT	Valine	35.1	13.1	2.7	0.01	−2.6	1.1	−2.4	0.02	−2.3	0.8	−2.7	0.01
MPNI-s17p	P23142	FBLN1	Valine	17.2	13.3	1.3	0.20	−2.1	1.1	−1.9	0.06	−1.1	0.8	−1.4	0.17
MPNI-s17p	Q12805	EFEMP1	Valine	21.7	12.6	1.7	0.09	−2.4	1.1	−2.2	0.03	−1.4	0.8	−1.8	0.08
MPNI-s17p	Q12860	CNTN1	Valine	28.4	14.4	2.0	0.05	−2.2	1.1	−2.0	0.04	−1.8	0.9	−2.0	0.04
MPNI-s17p	Q9NPH3	IL1RAP	Valine	10.1	12.9	0.8	0.43	−2.7	1.1	−2.4	0.02	−0.7	0.8	−0.9	0.39
MPNI-t12p	Q01459	CTBS	Valine	0.7	19.1	0.0	0.97	−3.8	1.6	−2.4	0.02	0.0	1.2	0.0	0.98
MPNI-t12p	Q01459	CTBS	3-hydroxybutyrate	0.5	6.0	0.1	0.94	−0.9	0.5	−2.0	0.05	0.1	0.4	0.1	0.89

Interactions between proteins and metabolites were tested for proteins identified in Table 2 with standardized coefficients above 3 in absolute value, and if the metabolite was previously associated with the mental health indicator. *FDR* False Discovery Rate. *se* standard error.

## Discussion

In this exploratory multi-omic study of mental health, we found 47 proteins significantly associated with depressive symptoms or the p-factor. Of these, 17 proteins were found to have more than one association across different ages, raters, and/or mental health indicators. Most protein associations were negative and involved the p-factor. We identified two protein–metabolite interactions associated with the p-factor, as well; these involved valine (metabolite) and proteins Mast/stem cell growth factor receptor Kit and Contactin-1. These associations are not indications of diagnostic markers nor are they suggested to be causative. However, these proteins and associations suggest plausible places to investigate biological pathways of interest in better understanding mental health disorders.

Those proteins associated with mental health across different ages (e.g., Contactin-1, Cadherin-13), different raters (e.g., IgGFc-binding protein, Complement C1q subcomponent subunit C), and/or different mental health indicators (e.g., Podocalyxin, Complement C4-B) may lead to fruitful in-depth investigations of biological mechanisms, given the greater consistency of those associations. Some of the proteins (e.g., Contactin-1, IgGFc-binding protein) found in this more extended, refined analysis overlap with a previous investigation [12], restricted to the p-factor only and using a different approach; that supports the stability of these associations. Particularly noteworthy is that several of the identified proteins were associated with both the p-factor and depressive symptoms. As such, these proteins may reflect underlying shared information between the p-factor and depressive symptoms, perhaps shared internalizing factors, since the p-factor aggregates internalizing factors along with externalizing factors (thought disorders are also included in the p-factor, but were not available in our dataset). Anxiety items and measures are not as abundant in our data, thus we did not include anxiety separately in this investigation; its inclusion could help clarify this overall p-factor–internalizing connection. However, phenotypic and genetic associations between externalizing factors and depression or depressive symptoms have been reported in the literature [18–20]. This suggests that the proteins associated with both the p-factor and depressive symptoms may not necessarily reflect only internalizing behaviors, but could potentially reflect a more complex picture that combines internalizing and externalizing factors.

Collectively, the proteins associated with mental health indicators in the current investigation point to key pathways that have been widely studied in the context of psychiatric disorders, with evidence suggesting their involvement. Among these, the interleukin-6 signaling pathway emerged with the highest degree of statistical significance in our analyses. Interleukin-6 is a cytokine, a signaling protein that regulates immune responses and inflammation in particular. Interleukin-6 has been shown to be consistently associated with depression [21], as well as with stress vulnerability [22] and suicidality [23]. Another major pathway we identified is the regulation of insulin-like growth factor (IGF) transport and uptake by IGF-binding proteins. IGFs are proteins with established links to psychiatric traits, and there is also growing evidence in the literature that this protein may also be associated with emotional and cognitive disorders [24]. The third most important pathway we identified in relation to identified proteins is that of post-translational protein phosphorylation. Protein phosphorylation is a post-translational modification (PTM) that occurs after a protein has been synthesized during translation. The role of PTMs in neuropsychiatric disorders has been relatively well studied in the literature, and in particular, several studies have demonstrated the role of protein phosphorylation in synaptic plasticity or learning ability [25, 26]. Overall, the results of the pathway analysis indicate that the proteins we identified, which are associated with mental health indicators spanning from childhood to adulthood, are implicated in well-established biological pathways linked to psychiatric disorders. This also suggests that key pathways associated with proteins linked to mental health throughout adolescence align with those reported in the literature, which primarily focuses on adults and animal models.

The most consistent protein–mental health associations may yield insights into enhanced biological understanding and improved treatment. For example, contactin-1 (associated with MPNI-pz, MPNI-s17p, MPNI-s14d, and has an interaction with valine) or its gene (CNTN1), involved in neuronal and oligodendrocyte development, has been shown to be associated with psychiatric illness (e.g., antidepressant treatment resistance, bipolar disorder) as well as neurodegenerative disease (e.g., Alzheimer’s and Parkinson’s) [27, 28]. Additionally, CNTN1 has been associated in the national FinnGen database (www.fingen.fi) with 50,000+ cases of depression medication use and “psychiatric diseases” among ethnic Finns [29]. In a recent large-scale proteomic analysis of older adults from the UK Biobank, the contactin-1 protein was also found to be negatively associated with depressive symptoms (log_10_(p) = 10.6) [13]. This suggests that contactin-1 is a good protein for future investigations of depression and related symptoms in all age groups.

Cadherin-13 (associated with GBI-22, MPNI-s14p, MPNI-s14d), a cell adhesion molecule, or its gene (CDH13) has also been shown in the literature to be associated with psychiatric illness (e.g., depression, substance abuse, attention-deficit/hyperactivity disorder) [30–33], as well as with psychiatric disorders among ethnic Finns in the FinnGen database [29]. Podocalyxin (associated with MPNI-s14p, MPNI-s14d), another protein involved in cell adhesion and transportation, or its gene (PODXL), has been shown to be involved with neural development, blood–brain barrier function, neuroinflammation, and neurodegenerative disease [34–36], as well as with neurological and psychiatric disorders among ethnic Finns in the FinnGen database [29]. The gene (FCGBP) coding for IgGFc-binding protein (associated with MPNI-pz, MPNI-s14p, MPNI-co14p), a protein involved in immune response, has been shown to be associated with bipolar disorder and depression [37, 38], as well as with neurological and psychiatric disorders among ethnic Finns in the FinnGen database [29]. Another protein involved in immunity, complement C1q subcomponent subunit C (associated with MPNI-s14p, MPNI-co17p) or its gene (C1QC) has been associated with depression [39–41], as well as with psychiatric disorders and depression among ethnic Finns in the FinnGen database [29]; furthermore, complement C4-B (associated with MPNI-s14p, MPNI-s14d) or its gene (C4B) is associated with white matter integrity, depression, and schizophrenia [42–44], as well as with psychiatric disorders, depression, and schizotypal disorders among ethnic Finns in the FinnGen database [29]. Lastly, mast/stem cell growth factor receptor Kit (associated with MPNI-s17p and valine interaction), a protein involved in cell signaling, or its gene (KIT), have been associated with obsessive compulsive disorder, schizophrenia, and autism spectrum disorders [45, 46], as well as being associated in the FinnGen database with psychiatric disorders, depression, anxiety disorders, and schizotypal disorders [29].

For mast/stem cell growth factor receptor Kit and contactin-1, a significant protein–metabolite interaction (metabolite: valine) was identified with the p-factor. While higher levels of contactin-1 or mast/stem cell growth factor receptor Kit were associated with higher p-factor scores at age 17, the effect sizes were reduced in participants with high levels of valine. To the best of our knowledge, these interactions have not previously been shown. Valine had previously been shown to be negatively associated with depressive symptoms, and approached significance with the p-factor [14], and contactin-1 has been shown to be associated with depression phenotypes in mice [47, 48] and older adults from the UK Biobank [13], as well as the previously mentioned psychiatric and neurodegenerative associations. Additionally, a study of essential amino acid (EAA) supplementation and protein expression in low physical activity older adults showed reduced expression of mast/stem cell growth factor receptor kit in relation to EAA supplementation [49]. Currently, however, the direct meaning of our found interactions cannot be clearly interpreted since how the two omic levels influence each other is poorly known. Additionally, our approach for testing for interactions was rather conservative in that we required independent association with both the metabolite (p < 0.05) and the protein (|standardized coefficient| > 3) before interaction testing, thus only 7 interactions were tested.

An additional point to raise with these findings involves the use of multiple raters and the frequent significant associations of proteins with self-reports of mental health. Self-reports from ages 14, 17, and 22 were among many of the significant associations with proteins. A couple of associations between proteins and mental health were even found with two different self-report measurements such as MPNI, CDI or GBI (e.g., Osteomodulin and Cadherin-13), while a couple of protein associations were found with adolescent ratings of both self-report and co-twin reports (e.g., IgGFc-binding protein and Complement C1q subcomponent subunit C). The effectiveness of adolescent self-reports to identify associations between mental health and metabolites [14, 15] or future psychiatric disorder [50] has previously been seen in our earlier studies, and others have also found adolescent self reports or ratings from other youth (e.g., peers) to also be important and unique characterizations of mental health, possibly because they offer a view across environments such as home, school and leisure activities [51, 52]. However, some studies, for example on ADHD, have not seen this same pattern [53]. In general though, it seems that adolescent self-reports for metabolomic and proteomic associations should be considered potentially valuable, and used at least along with other raters in future studies. While reports from parents or teachers may accurately reflect external perceptions of depressive symptoms or aggressive behavior, self-reports may be more reflective of the individual’s overall health. Additional investigations focusing on differences in proteomic associations across raters and different contexts may pave the way to a better understanding of protein functions in dimensions of mental health.

Our study has many strengths including using multiple omics levels, mental health indicators, and raters of mental health, but it is also important to consider its limitations. For example, our proteomics panel (and metabolomics panel) included only a portion of the entire proteome (and metabolome), thus as panels with a higher number of proteins become available, extending these analyses would be important. Additionally, despite not looking at the entire proteome, correcting for multiple testing limited our ability to identify associations and interactions. Another limitation is that we did not examine the role of medication use or substance use in confounding associations between plasma proteins and mental health indicators. A recent large-scale study identified proteins associated with medication use and smoking, suggesting that these variables influence the proteome [13]. In the current study, we found no significant association between depression medication use reported at the time of blood collection and plasma proteins (number of anti-depressant users: 20 out of 730), suggesting that anti-depressants may play a minor role in confounding the reported associations. Lastly, since we had only proteomic and metabolomic data available at one time point (age 22), we are unable to draw any causal conclusions. We used both mental health indicators at the same time point as the blood draw as well as earlier, not to suggest causal pathways, but to indicate whether patterns of associations were consistent, as in our previous investigations [14, 15]. To investigate causality, one could use Mendelian randomization [54] or collect and investigate longitudinal changes in mental health and omics data. Longitudinal data could enhance understanding of omic profiles of those with persistent poor mental health and/or those proteins that are sensitive to fluctuations in mental health over short- or mid-term time periods.

In conclusion, this study investigated proteomic associations between depressive symptoms and the p-factor using multiple mental health indicators and raters. It may be the first to identify a protein–metabolite interaction between two proteins (Mast/stem cell growth factor receptor Kit and Contactin-1) and valine (metabolite) in relation to p-factor levels. Most proteins were associated with the p-factor or both the p-factor and depressive symptoms, and several of the significant proteins have been associated with functions and disorders in the brain or are involved in immunity or inflammation. Results add to the newly emerging list of possible biological molecules and pathways to investigate the etiology, severity, and treatment options in mental health disorders.

## Methods

### Cohort and participants

The FinnTwin12 cohort yielded the data for this investigation. FinnTwin12 is a Finnish population-based cohort of twins born 1983–1987 with data collected from parents, teachers, classmates, and twins themselves at ages 11/12, 14, 17, and approximately 22 years old [55]. Data collection involved several questionnaires over the different waves of collection; at age 14 and 22, an intensively studied subset of twins underwent semi-structured psychiatric interviews, additional questionnaires, and collection of biological samples.

Only individuals for whom plasma proteomics were obtained (from the intensive subset) were selected for the present analysis. Pregnant women (n = 53) and those taking cholesterol medication (n = 1) were also excluded, leading to a final sample size of N = 730 twins. This sample comprised 56% females, and participants were 22.3 years old on average at the time of blood sampling (sd: 0.6; range: 21.0–25.0).

For each of the first three data collection waves, BMI was calculated from self-reported height and weight. In the wave corresponding to age 22 years, we calculated the BMI from the height and weight measurements that were clinically assessed on the day of the visit for the blood sample. Missing BMI information for each wave did not exceed 10% (range: 0–9.9%) and represented 4.8% of all BMI measures available. Missing BMI information was therefore imputed using the median BMI of each wave. Mean BMI was 17.6 (sd = 2.6), 19.4 (sd = 2.7), 21.4 (sd = 2.7) and 23.3 (sd = 4.0) kg.m^−2^ at ages 12, 14, 17 and 22, respectively.

Ethical approval for all data collection waves was obtained from the ethical committee of the Helsinki and Uusimaa University Hospital District and Indiana University’s Institutional Review Board. All study methods were performed in accordance with the relevant guidelines and regulations. At ages 12 and 14, parents of the twins provided consent for the twins’ participation, while the twins themselves provided written consent at ages 17 and 22.

### Questionnaires and indicators of mental health

At age 12, 14, and 17 data collection waves, different raters were asked to fill out the modified Multidimensional Peer Nomination Inventory (MPNI) questions regarding the twins [56, 57]. Raters included parents and teachers at age 12, teachers, the twins themselves and their co-twin at age 14, and the twins themselves and their co-twin at age 17. The MPNI can be used at multiple subscale and dimensional levels, and captures general levels of emotional and behavioral problems. Subscales include depression and social anxiety (these two are part of the internalizing dimensional scale), and aggressive behavior, hyperactivity/impulsivity, and inattention (these three comprise the externalizing dimensional scale). Additionally, the internalizing and externalizing problem dimensions can be summarized into a p-factor scale (thought disorder symptoms were not collected) [14]. A mean score is used, allowing no missing values for subscales and 4 missing values for the p-factor scale (missing items imputed to the mean score). Additionally, a “combined” p-factor score was created using the p-factor scores of all seven of the available MPNI ratings (Cronbach’s alpha=0.76), because we know that ratings from different raters are not highly correlated [15, 58], even though factor analysis suggested a unique factor to be retained. For this combined p-factor, we therefore averaged the seven individual p-factors to capture shared variability across different p-factors, which we previously scaled [12] to mean zero and variance one.

At the age 14 and 22 data collection waves, semi-structured psychiatric interviews (Semi-Structured Assessment for the Genetics of Alcoholism; SSAGA [59]) were conducted which included DSM-IV (Diagnostic and Statistical Manual of Mental Disorders) criteria used to create psychiatric diagnoses, including MDD. For the age 14 assessment, we used the number of lifetime depressive symptoms [60] and for age 22 MDD diagnosis (Table 1). The choice of using lifetime depressive symptoms rather than MDD diagnosis at age 14 was made because only 3 participants were diagnosed with MDD, whereas 120 participants reported having one or more depressive symptoms during their lifetime. In contrast, 103 participants were diagnosed with MDD at age 22. In the SSAGA, medication use (including anti-depressant medication use) data was also collected.

At age 17 and 22 data collection waves, the twins filled out the General Behavior Inventory (GBI) questionnaire [61, 62]. This self-rated modified depression questionnaire includes a validated 10 items (the original GBI has 73 items with multiple subscales). A sum score is used, with one missing value allowed.

Lastly, at the age 14 data collection wave, twins filled out the Children’s Depression Inventory (CDI [63]), a 27-item inventory assessing depressive symptoms in youth. Each question was associated with 3 responses that reflected the severity of the symptoms associated with depression and were coded as 0, 1, or 2. The CDI score was defined as the sum of these scores, thus ranging from 0 to 54, with higher scores referring to higher levels of depressive symptoms. A maximum of 2 missing responses was allowed (59 and 19 participants had 1 or 2 missing items, respectively), and missing values were imputed with zeros, i.e., we did not increase the CDI score when missing values occurred, because missing values were not guaranteed to be randomly distributed.

In total, 21 indicators of mental health were used: MPNI parent age 12 depression (MPNI-p12d), MPNI teacher age 12 depression (MPNI-t12d), MPNI-t14d, MPNI self rating age 14 depression (MPNI-s14d), MPNI co-twin rating age 14 depression (MPNI-co14d), MPNI-s17d, MPNI-co17d, MPNI parent age 12 p-factor (MPNI-p12p), MPNI-t12p, MPNI-t14p, MPNI-s14p, MPNI-co14p, MPNI-s17p, MPNI-co17p, MPNI p-factor summary z-score (MPNI-pz), MDD age 14 (symptom count; MDD-14), MDD-22 (yes/no), depression medication use age 22 (yes/no; medication-22d), GBI age 17 (GBI-17), GBI-22, and CDI age 14 (CDI-14). Descriptive summaries of the 21 mental health indicators regarding Ns, means, standard deviations, ranges, skewness, and kurtosis can be found in Table 1. Additionally, correlations between continuous mental health indicators are shown in Fig. 2A, B as both a network (A: abs(cor) > 0.2) or correlation matrix (B).

### Omics processing

At the age 22–year wave, blood plasma samples were collected after overnight fasting and a request to abstain from alcohol and tobacco from the night before sampling. In 2010, the samples were processed to obtain metabolomics data at Nightingale (formerly, Brainshake). In 2022, the samples were processed to obtain proteomics data at the Turku Proteomics Facility (Turku Proteomics Facility, Turku, Finland). Further details of proteomic and metabolomic data processing are described in the corresponding subsections.

#### Proteomic data processing

Proteins from plasma samples of the 730 individuals used in this study were processed in four batches at the Turku Proteomics Facility (Turku Proteomics Facility, Turku, Finland) using their LC-ESI-MS/MS (Q Exactive HF mass spectrometer) proteomics platform; a detailed description of the data is provided elsewhere [12]. Proteins were subjected to precipitation and in-solution digestion in accordance with the standard protocol of the Turku Proteomics Facility. A commercial kit (High Select™ Top14 Abundant Protein Depletion Mini Spin Columns, cat. No: A36370, ThermoScientific) was used to deplete the most abundant proteins from plasma prior to proteomic analysis. The data were first analyzed using Spectronaut software and included local normalization of the data [64]. Raw data was processed as described in detail elsewhere [10]. Briefly, data processing included log_2_ transformation of protein values, assessment of outliers, exclusion of proteins with >10% missing values, imputation of missing values using the lowest observed value per batch, and corrections for batch effects using Combat [65]. The protein abundances were scaled such that one unit corresponded to one standard deviation with a mean of zero. The final proteomic dataset consisted of 439 proteins for the 730 individuals in the study.

#### Metabolomic data processing

Metabolites from plasma samples of the 730 individuals used in this study were processed in one batch at Nightingale (formerly, Brainshake) using their automated high-throughput ^1^H nuclear magnetic resonance spectroscopy (NMR) metabolomics platform [14, 15]. Metabolite values were available in mmol/l, and were log transformed. We focused on three specific metabolites in this study that had already been associated in FinnTwin12 with mental health: 3-hydroxybutyrate was negatively associated with aggressive behavior and the p-factor [14, 15] and valine and leucine were negatively associated with depression [14].

### Statistical analyses

We first quantified the associations between protein levels and mental health indicators using linear and generalized mixed-effects models. We successively modeled each mental health indicator (n = 21) as a dependent variable while proteins were used as independent variables. Age at blood sampling, sex and BMI at blood sampling were used as covariates. In models where mental health indicators were derived from questionnaires completed at ages 12, 14 or 17, BMI at 12, 14 or 17 years of age, respectively, was also included as a covariate. The inclusion of adolescent BMI was performed because the variability of BMI at blood sampling was not found to be strongly determined by self-reported BMI during adolescence, since the coefficients of determination (R-square) derived from univariate linear models were in the range of 37–52%. This ensured identification of proteins associated with adolescent mental health independent of both adolescent and adult BMI. For models using the z-scored p-factor, given that the p-factor was averaged across different ages, we did not include BMI measurements assessed during adolescence but only BMI at blood sampling. To correct for family relatedness in the data, we used family identifiers as random effects. Depending on whether the mental health indicator assessed was a continuous or binary outcome, we used linear and generalized mixed-effects models, respectively. Nullity of fixed-effect coefficients related to proteins was tested, from which nominal p-values were derived. For each set of associations between a mental health indicator and the 439 proteins, we used the Benjamini–Hochberg procedure to control the False Discovery Rate (FDR; α = 0.20).

Following the identification of proteins associated with mental health indicators, we sought to explore the molecular determinants that may drive these associations by conducting a pathway analysis. We used the Reactome pathway database [66] with the list of unique protein UniProt IDs that we identified to be associated with mental health indicators used as input. We reported the results of the pathway analysis.

Finally, we sought to determine whether, when significant associations between protein levels and mental health indicators were identified, the addition of interaction terms with metabolites reported in the literature could deepen our understanding of the molecular basis of mental health (Fig. 1). We carried out interaction analyses by adding fixed-effect coefficients for the metabolite and its interaction with the protein to the initial model(s) described above. Due to limited statistical power to test for interactions between proteins and metabolites, we restricted interaction analyses to proteins that were strongly associated with mental health indicators, i.e., with a standardized coefficient above 3 in absolute value (Fig. 1). In addition, interaction analyses were performed if a metabolite was found to be sufficiently associated with the mental health indicator to be modeled (nominal p < 0.05), as assessed by initial linear or generalized mixed effects models described above. Interaction nullity was assessed by t- or z-test, and interactions were considered significant if nominal p-values were below 0.05. Models were run using the R package lme4 version 1.1-30 under the R Studio environment (version 4.1.3).

### Data access

The data used in the analysis is deposited in the Biobank of the Finnish Institute for Health and Welfare (https://thl.fi/en/web/thl-biobank/forresearchers). It is available to researchers after written application and following the relevant Finnish legislation.

## Supplementary information

Table S1: Associations between protein abundance and mental health indicators with nominal p-value under 0.05

Table S2: Pathway analysis of proteins associated with mental health indicators
